# Fingolimod Attenuates Splenocyte-Induced Demyelination in Cerebellar Slice Cultures

**DOI:** 10.1371/journal.pone.0099444

**Published:** 2014-06-09

**Authors:** Adam J. Pritchard, Anis K. Mir, Kumlesh K. Dev

**Affiliations:** 1 Drug Development, School of Medicine, Trinity College Dublin, Dublin, Ireland; 2 Autoimmunity, Transplantation and Inflammatory Disease, Novartis Institutes for BioMedical Research, Novartis Pharma AG, Basel, Switzerland; Hannover Medical School, Germany

## Abstract

The family of sphingosine-1-phosphate receptors (S1PRs) is G-protein-coupled, comprised of subtypes S1PR1-S1PR5 and activated by the endogenous ligand S1P. The phosphorylated version of Fingolimod (pFTY720), an oral therapy for multiple sclerosis (MS), induces S1PR1 internalisation in T cells, subsequent insensitivity to S1P gradients and sequestering of these cells within lymphoid organs, thus limiting immune response. S1PRs are also expressed in neuronal and glial cells where pFTY720 is suggested to directly protect against lysolecithin-induced deficits in myelination state in organotypic cerebellar slices. Of note, the effect of pFTY720 on immune cells already migrated into the CNS, prior to treatment, has not been well established. We have previously found that organotypic slice cultures do contain immune cells, which, in principle, could also be regulated by pFTY720 to maintain levels of myelin. Here, a mouse organotypic cerebellar slice and splenocyte co-culture model was thus used to investigate the effects of pFTY720 on splenocyte-induced demyelination. Spleen cells isolated from myelin oligodendrocyte glycoprotein immunised mice (MOG-splenocytes) or from 2D2 transgenic mice (2D2-splenocytes) both induced demyelination when co-cultured with mouse organotypic cerebellar slices, to a similar extent as lysolecithin. As expected, *in vivo* treatment of MOG-immunised mice with FTY720 inhibited demyelination induced by MOG-splenocytes. Importantly, *in vitro* treatment of MOG- and 2D2-splenocytes with pFTY720 also attenuated demyelination caused by these cells. In addition, while *in vitro* treatment of 2D2-splenocytes with pFTY720 did not alter cell phenotype, pFTY720 inhibited the release of the pro-inflammatory cytokines such as interferon gamma (IFNγ) and interleukin 6 (IL6) from these cells. This work suggests that treatment of splenocytes by pFTY720 attenuates demyelination and reduces pro-inflammatory cytokine release, which likely contributes to enhanced myelination state induced by pFTY720 in organotypic cerebellar slices.

## Introduction

The family of sphingosine 1-phosphate receptors (S1PRs) are G-protein coupled and designated as receptor subtypes 1-5 (S1PR1-5) [1]–[5]. These receptor, in particular the S1PR1 subtype, have been described over the last decade as important modulators of immune cell migration [6]. Recently, the orally available S1PR agonist, fingolimod (FTY720), has shown efficacy in the treatment of relapsing remitting multiple sclerosis, supporting the use of S1PRs as *bona fide* drug targets [7]. While the endogenous sphingolipid ligand, S1P, is phosphorylated from sphingosine by sphingosine kinase 1 (SphK1) and 2 (SphK2) [8], [9] the ‘pro-drug’ FTY720 is phosphorylated, primarily by SphK2, to its active form phosphate-FTY720 (pFTY720) [10]–[12]. In its phosphorylated form, pFTY720 is a full agonist of S1PR1, R4 and R5, as well as being a partial agonist of S1PR3, but displays no affinity for S1PR2 [12]. It has been suggested that pFTY720 internalises S1PR1s to cause sequestration of T cells within the lymph nodes [6], [13] likely preventing S1P-dependent T cell transmigration into the peripheral circulation and consequently into the central nervous system (CNS). Notably, however, reports investigating the effects pFTY720-mediated internalisation of S1P1Rs on the activation state of the lymphocytes or related cytokine release require further elucidation. For example, some studies show that a subpopulation of regulatory T cells (Tregs) may be functionally augmented by pFTY720 [14]–[17], which has been suggested as potentially beneficial in autoimmune or inflammatory illnesses. In contrast, others put forward the idea that pFTY720 may prevent the proliferation of Tregs and functionally impair them [18]. Thus, further studies examining the effects of pFTY720 on these T cell subpopulations may prove useful.

There is now a growing body of evidence to support that S1PRs also play a number of roles in regulating the physiology of neuronal and glial cells in the CNS [19]. With regard to oligodendrocyte function and myelination state, many studies have reported the positive effects of pFTY720 on both these processes, where S1PRs are suggested to play roles in remyelination as well demyelination [20]. The first of these studies elegantly described how pFTY720 increased remyelination 14 days after lysolecithin (LPC)-induced demyelination, which was suggested to be driven via S1P3R/S1P5R, with S1P1R limiting remyelination [21]. We then showed, using rat organotypic cerebellar slice cultures, that pFTY720 and SEW2871 (a S1PR1-specific agonist) also inhibited LPC-induced demyelination as assessed by myelin basic protein (MBP) immunofluorescence [22]. In that study, we reported both pFTY720 and SEW2871 inhibited the release of several chemokines in conditions of LPC-induced demyelination, including LIX (CXCL5), MIP-1alpha, and MIP-3alpha [22]. It was noteworthy, at that time, we also observed that the organotypic slice cultures stained positive for a number of immune cells [22], as previously reported by others [23], [24]. This finding raised the question whether pFTY720 attenuated demyelination by reducing pro-inflammatory response of these ‘brain-slice resident’ immune cells and/or by directly altering neuronal and/or gial cell function.

One of the challenges of investigating the role of S1PRs in oligodendrocytes on myelination has been the limitation to assess oligodendrocyte function in the context of inflammatory models. The specific knockout of S1PR1 from oligodendrocytes appears to increase sensitivity to cuprizone-induced demyelination [25], although no deficits in myelination state has been reported for S1PR5-null mice [26]. In cuprizone and LPC models of demyelination, FTY720 has not been shown to rescue myelination state, although FTY720 has been shown to attenuate cuprizone-induced damage to oligodendrocytes in the corpus callosum [25], [27]. Similar to the reported *in vitro* studies [22], [28], these protective effects of FTY720 are associated with a reduction in pro-inflammatory cytokines and chemokines [25]. From these studies, it appears that pFTY720 promotes myelin repair by likely modulating both S1PRs expressed in immune and glia cells, although the relative contribution of each cell type remains to be fully established. Moreover, to our knowledge, the effect of pFTY720 on peripheral immune cells that have already transmigrated into the CNS, prior to treatment, has not been well established. Here, in a first attempt to answer this question and to use an experimental model not dependent on chemical-induced demyelination, we have now investigated the effects of pFTY720 on splenocyte -induced demyelination directly applied to cerebellar brain slice cultures.

## Materials and Methods

### Ethics Statement

C57BL/6 mice (Harlan UK) were maintained in the BioResources unit at Trinity College, Dublin, Ireland under veterinary supervision throughout the study. Myelin oligodendrocyte glycoprotein (MOG) peptide-immunisation of C57BL/6 mice was performed under a license issued by the Department of Health (Ireland) in accordance with the guidelines laid down and approved by Trinity College Dublin ethical committee (IACUC). All other animal work fell under schedule 1 guideline where animals, without any *in vivo* manipulation, were sacrificed by cervival dislocation in isolation from other animals. Animals were housed in a temperature-controlled room under illumination with a 12 h light:12 h dark cycle (lights on from 06h00 to 18h00) and both food and water were available ad libitum.

### In vivo MOG-immunisation of C57BL/6 mice

Female C57BL/6 mice (8-10 week old) were sub-cutaneously injected at the base of the spine with 200 µl of myelin oligodendrocyte glycoprotein peptide (MOG_35-55_, AnaSpec) at a concentration of 500 µg/ml in Complete Freund's Adjuvant (Chondrex). Mice were also administered an interperitoneal injection of 200 µl pertussis toxin (PTX) at 2.5 µg/ml on day 0 and again on day 2. An interperitoneal injection (i.p.) of FTY720 (2-amino-2-[2-(4-octylphenyl)ethyl] propane-1,3-diol; obtained from Novartis Pharma, Basel, Switzerland) was given at 8 µg/day (corresponding to 0.3 mg/kg/day, with animal weight of 35 g mouse as described previously [12]) for 10 days (days 0-9). Spleens were taken on day 10 after immunisation.

### In vitro culture and stimulation of MOG reactive splenocytes

Spleens were isolated from control C57BL/6 mice, MOG-immunised C57BL/6 mice or 2D2 transgenic mice [29] (kindly provided by Dr. Denise Fitzgerald, Queen's University, Belfast). The MOG-immunisations were conducted in three separate experiments, with 6 mice per group in each experiment. The groups (6 mice per group) were naive mice (non-immunised), MOG-immunised mice administered i.p. with saline vehicle control, and MOG-immunised mice administered i.p. with FTY720. A single cell suspension was created from isolated spleens, erythrocytes were lysed and cells were cultured in RPMI-1640 media supplemented with 10% FCS, 1% L-glutamine, 1% penicillin/streptomycin and 0.1% β-mercaptoethanol (R-10). MOG reactive splenocytes from MOG-immunised mice or 2D2 transgenic animals (2×10^6^ cells/ml) were incubated with 25 µg/ml MOG_35-55_ peptide for 48 hrs with or without pFTY720 (1 nM-1 µM) (obtained from Novartis Pharma, Basel, Switzerland). The pFTY720 was prepared as a 10 mM stock in DMSO and vehicle control concentrations for *in vitro* studies were 0.00001%–0.01% DMSO for 1 nM-1 µM pFTY720, respectively. Cells from control mice (2×10^6^ cells/ml) were stimulated with plate-bound mouse anti-CD3 and anti-CD28 (BD Biosciences) at 1 µg/ml for 48 hrs. For ELISA and FACS studies, cells were activated as above with or without pFTY720 (1 nM-1 µM), TGF-β (5 ng/ml) and/or IL-2 (20 ng/ml) for four days at 37°C and 5% CO_2_ before removal of media for ELISA and staining of cells for FACS analysis.

### Culture of Organotypic Cerebellar Slices

Experiments were conducted using tissue isolated from postnatal day 10 (P10) C57BL/6 mice in accordance with EU guidelines and protocols approved by the Trinity College Dublin ethics committee. The cerebellar slice culture was based on published protocols (Birgbauer et al., 2004). Briefly, 400 µm parasagittal slices of cerebellum were cut using a McIlwain tissue chopper. The slices were prepared from litters of 4–6 pups and randomly distributed between conditions and 5–6 slices were grown on each cell culture insert (Millicell PICMORG50). Slices were cultured using an interface method with 1 mL of medium per 35 mm well. For the first 3 days *in vitro* (DIV), slices were grown in serum-based medium (50% Opti-Mem, 25% Hanks' buffered salt solution (HBSS), 25% heat-inactivated horse serum and supplemented with 2 mM Glutamax, 28 mM D-glucose, 100 U/mL penicillin/streptomycin and 25 mM HEPES) at 35.5C and 5% CO2. After 3 DIV, slices were transferred to serum-free medium (98% Neurobasal-A and 2% B-27 (Invitrogen), supplemented with 2 mM Glutamax, 28 mM D-glucose, 100 U/mL penicillin/streptomycin and 25 mM HEPES). The slices were transferred to serum free media to reduce the possible effects of S1P present in serum, on S1PRs during the experiment. To induce demyelination, 12 DIV cultures were transferred to fresh serum-free medium ready for treatment. Thus, demyelination was induced at 12 DIV and examined at 14 DIV.

### Co-culture of Splenocytes and Organotypic Cerebellar Slices

Organotypic cerebellar slices were cultured according to the method described above. Cultured spleen cells were counted and washed by centrifugation before being resuspended at a concentration of 1×10^6^ cells/ml in organo-serum media (50% Optimem, 25% Hank's Buffered Salt Solution, 25% Heat inactivated horse serum, 1.1% D-glucose (27.5 mM), 1.1% Glutamax (2 mM), 1% Penicillin (10,000 U/ml) and Streptomycin (100 U/ml) (Pen/Strep) and 1% HEPES (10 mM)). The cells were washed again by centrifugation and the resuspended splenocyte cell suspension (5 µl, ∼5×10^4^cells) was added directly onto each slice and co-cultured for 48 hrs at 35.5°C and 5% CO_2_. As a positive control, slices were treated with 0.35 mg/ml lysolecithin (LPC: Sigma, L-4129) for 18 hrs at 35.5°C and 5% CO_2_ before being moved to fresh media for the remainder of the 48hr treatment.

### Biochemical Analysis

For flow cytometry, cells were washed in PBS supplemented with 1% FCS, 1% Sodium Azide and 3 mM EDTA (FACS buffer) and stained with a cocktail of anti-CD3 PE, anti-CD4 APC-Cy7 and anti-CD25 FITC (eBioscience). Cells were then fixed and permeabilized using eBioscience FoxP3 staining kit (77-5775-40) and stained with anti-Foxp3 APC. Samples were examined using a BD LSRFortessa flow cytometer and analyzed using FlowJo software (Treestar). For ELISA, supernatants from cell culture were removed and examined for their cytokine content using ELISA kits for IL10 (DY417) and IFNγ (DY485) according to the manufacturer's instructions (R&D systems). For Western blotting, cerebellar slices were scraped from the culture membrane and suspended in PTxE buffer (PBS, 1% Triton-x, 1 mM EDTA) using mechanical homogenisation and sonication. Western blotting was performed on tissue suspensions as we have described previously [30]. Primary antibodies used were: anti-MOG (Millipore, MAB5680), anti-myelin basic protein (MBP) (Abcam ab40390), anti-actin (ECM Biosciences, AM2021), anti-neurofilament H (NFH) (1/1000 dilution: Millipore, MAB5539). Secondary antibodies used were: HRP conjugated mouse (Sigma, A8924), and HRP conjugated rabbit (GE Healthcare, NA934).

### Immunocytochemistry

Immunostaining and analysis was performed as we have described previously [22]. Confocal images were captured using a LSM 510 Meta microscope at 20× or 40× magnification. Image stacks were initially used to analyse all layers of the slice and showed that optimal staining was confined to the central layers; images were thus taken from central layers within the tissue. These resulting images were analysed using EBImage software and run through the statistical programming environment, R as we have described previously [22]. In brief, EBImage allows analysis of multiple colour channels separately, where every pixel (1024×1024) is assigned an intensity value between 0 and 1, where 0 represents no intensity and 1 represents an intensity value of 4096 as per a 12 bit image. In this case, NFH staining (red) was used as a “mask” in order to measure the amount of MBP staining (green) co-localized with NFH only, i.e., neuronal MBP staining. The percentage of green pixels (MBP) which were co-localized with red (NFH) was calculated. This co-localisation of MBP and neurofilament-H (NFH) staining was quantified and described as the level of myelination. Notably however this measure does not indicate the presence of compact myelin. Oligodendrocytes in vitro can express MBP even in the absence of axons to myelinate. Thus, the overlap of MBP and NFH likely indicates that the oligodendrocytes may be wrapping axons, but is not proof-positive for the presence of compact myelin [31], [32]. Primary antibodies used were as above. Secondary antibodies used were: DyLight 549 conjugated anti-chicken (1/1000: Jackson Immunoresearch), DyLight 488 anti-rabbit (1/1000: Jackson Immunoresearch).

### Statistical Analysis

All statistical analysis was performed using Prism 5 GraphPad Software package. The normality of the data was established by Bartlett's test for equal variances and a one-way ANOVA with Newmann-Keuls post-hoc test was used to compare groups. Individual statistical tests are described in text and figure legends. The significance levels (or alpha levels) were set at p<0.05*, p<0.01** and p<0.001***.

## Results

### Splenocytes isolated from MOG-immunised mice induce demyelination

**Figure 1:**
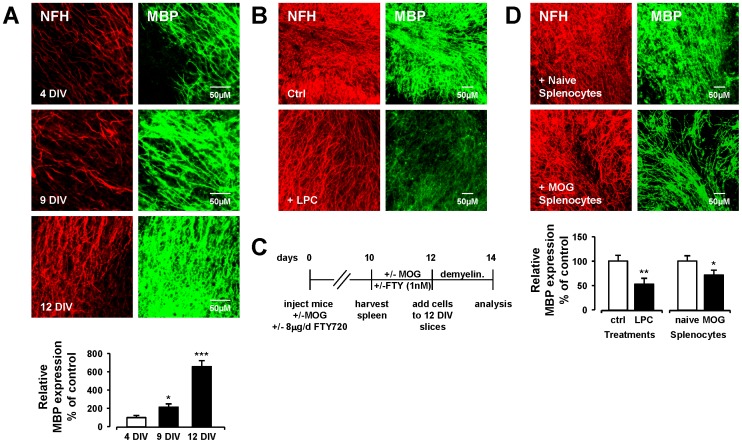
MOG reactive splenocytes induced demyelination in organotypic cerebellar slice cultures. (**A**) Time dependent myelination. Representative confocal images displaying MBP (MBP, green) and neurofilament (NFH, red) immunoreactivity at 4, 9 and 12 DIV. Confocal images captured at ×40 magnification. Bar graph illustrates relative MBP immunoreactivity (i.e. co-localization of MBP and neurofilament) in cerebellar slices compared to 4 DIV (100% as control) expressed as averages +/− SEM (12 slices per treatment group). (**B**) LPC-induced demyelination. MBP expression is reduced in cerebellar slices treated with LPC (350 µg/ml) as previously reported [22]. (**C**) Experimental setup. MOG-immunisation procedure and slice/splenocyte co-culture timeline is shown. (**D**) MOG reactive splenocytes induce demyelination. MBP expression is reduced in cerebellar slices treated with MOG reactive splenocytes (i.e. splenocytes isolated from MOG-immunised mice and re-stimulated *in vitro* with 25 µg/ml MOG), but not naive splenocytes isolated from control mice. Bar graph illustrates relative MBP immunoreactivity (i.e. co-localization of MBP and neurofilament) in cerebellar slices compared to no treatment (100% as control) expressed as averages +/− SEM (18–24 slices per treatment group). Significant difference *p<0.05, **p<0.01 and ***p<0.001 from control; One-way ANOVA and Newman-Keuls post-hoc test.

Prior to examining the effects of S1PR modulation on splenocyte-induced demyelination, the development and organization of neuronal fibers and myelin under control conditions, as determined by NFH and MBP expression, respectively, was examined in mouse organotypic cerebellar slice cultures. While we describe the co-localization of MBP and NFH staining as the level of myelination, it should be noted however that this measure does not indicate the presence of compact myelin. Oligodendrocytes in vitro can express MBP even in the absence of axons to myelinate. Thus, the overlap of MBP and NFH likely indicates that the oligodendrocytes may be wrapping axons, but is not proof-positive for the presence of compact myelin [31], [32]. An approximate 2-fold increase in myelination between day 4 *in vitro* (DIV4) and DIV9 (p<0.05, one-way ANOVA and Newmann-Keuls post-hoc test) was found (**Figure 1A**). Myelination continued beyond DIV9, where an approximate 6-fold increase in MBP expression was found at DIV12 compared to DIV4 (p<0.001, one-way ANOVA and Newmann-Keuls post-hoc test). At this later time point, MBP and neurofilament staining was found pre-dominantly colocalised along the Purkinje cell axons, indicative of axons displaying an intact myelin sheath (**Figure 1A**). Based on this information, co-culture of slices and splenocytes was initiated at DIV14. As control, we found slices treated with LPC used at a concentration and time similar to previous reports by our group and others (350 µg/ml for 18 h) [21], [22] induced a significant demyelination (52.7%+/−12.6%) compared to control (p<0.01, one-way ANOVA and Newmann-Keuls post-hoc test) confirming our previous findings [22] (**Figure 1B**). Next splenocyte-induced demyelination was examined in these organotypic cerebellar slice cultures. We have recently shown that naïve cells isolated from peripheral lymph nodes of wild-type mice are not detrimental to organotypic hippocampal slice cultures, and indeed are neuroprotective against toxicity induced by kainate- and oxygen-glucose deprivation [33]. Accordingly, naïve-splenocytes did not induce any significant change in MBP staining when applied to organotypic cerebellar slice cultures (**Figure 1D**). In contrast, splenocytes isolated from spleen of MOG-immunised mice (MOG-splenocytes), re-stimulated *in vitro* with MOG_35-55_ (25 µg/ml for 48 h) and co-cultured with cerebellar slices for 48 h (**Figure 1C**) induced a significant decrease in MBP staining (59.2%+/−8.0%) compared to naïve-splenocytes (p<0.05, one way ANOVA and Newmann-Keuls post-hoc test) (**Figure 1D**). Overall, these results support our data and data published by others, which demonstrate the amount of demyelination induced by MOG-splenocytes is comparable to that seen in LPC treated slices (**Figure 1D**).

### In vivo and in vitro treatment with FTY720 attenuates demyelination induced by MOG-splenocytes

**Figure 2:**
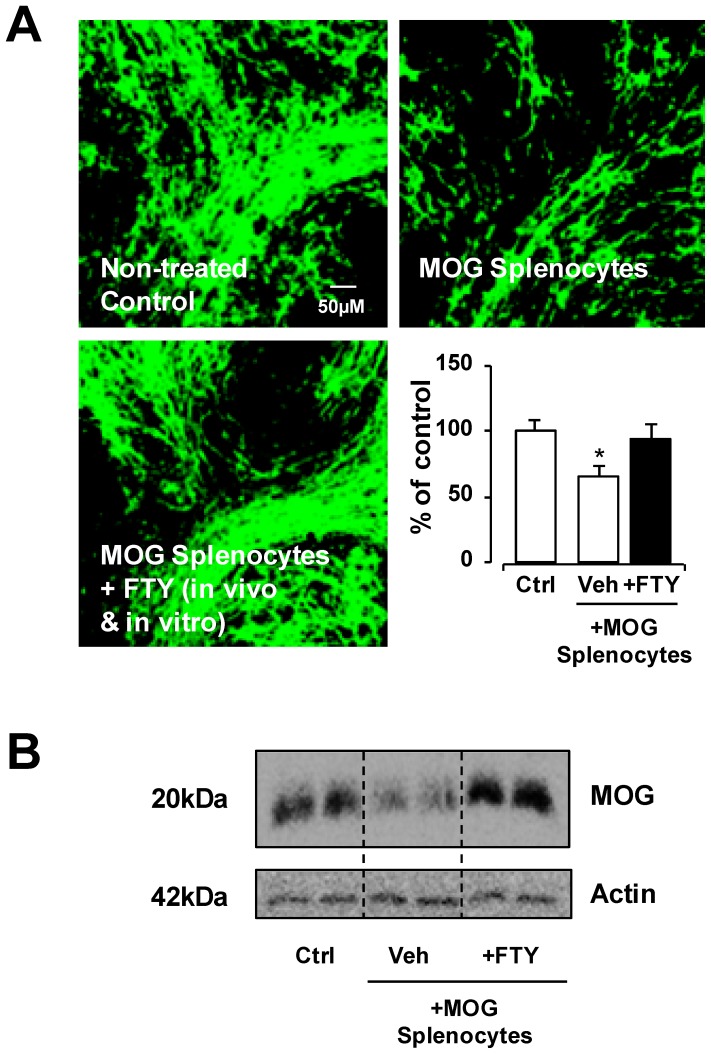
Treatment of pFTY720 in vivo and in vitro reduces MOG reactive splenocyte-induced demyelination isolated from MOG-immunised mice. (A) MOG reactive splenocytes (i.e. splenocytes isolated from MOG-immunised animals and re-stimulated *in vitro* with 25 µg/ml MOG) reduced MBP staining in cerebellar slices. MOG reactive splenocytes isolated from MOG-immunised animals treated with pFTY720 (8 µg/day, for 10 days) *in vivo* and treated again with pFTY720 (1 nM) during re-stimulation *in vitro* with 25 µg/ml MOG prevented demyelination caused by these isolated cells. Bar graph illustrates relative MBP immunoreactivity (i.e. co-localization of MBP and neurofilament) in cerebellar slices compared to no treatment (100% as control) expressed as averages +/− SEM (20 slices per treatment group). There was a significant decrease of MBP staining in slices co-cultured with vehicle treated MOG reactive splenocytes compared to control slices (*p<0.05), which was reduced by pFTY720 treatment; One-way ANOVA and Newman-Keuls post-hoc test. (B) Western blot shows decreased levels of MOG protein in MOG reactive treated slices, with *in vivo*/*in vitro* pFTY720 treatment reducing demyelination.

Reports have shown that FTY720 can reduce the pro-inflammatory response of immune cells or induce anti-inflammatory cell phenotypes [15]. As a positive control and to further confirm the validity of this splenocyte-induced demyelination model, the ability of FTY720 in attenuating demyelination induced by MOG-splenocytes was examined. The MOG-immunised mice were co-treated with FTY720 *in vivo* (8 µg/mouse/day for 10 days) and isolated MOG-splenocytes were also treated with pFTY720 *in vitro* (1 nM for 48 h) during re-stimulation with MOG_35-55_ peptide (25 µg/ml for 48 h) before co-culture with organotypic cerebellar slices. Confocal images showed significant reduction in MBP expression in slices co-cultured with vehicle-treated MOG-splenocytes (65.2%+/−7.9%), compared to untreated slice control (p<0.05, one way ANOVA and Newmann-Keuls post-hoc test). Not surprisingly, MOG-splenocytes treated with FTY720 (94.0%+/−11.1%) induced no difference in MBP expression compared to untreated slice control (**Figure 2A**). In addition, Western blotting for MOG protein showed a decrease in MOG expression in cerebellar slices co-cultured with vehicle-treated MOG-splenocytes compared to untreated slice control and as expected FTY720 attenuated the effects of MOG-splenocytes (**Figure 2B**). These data indicate that *in vivo* treatment of MOG-immunised mice with FTY720 followed by *in vitro* treatment of MOG-splenocytes with pFTY720 reduces the ability of these MOG-splenocytes to induce demyelination.

### In vitro treatment of MOG-splenocytes with pFTY720 is sufficient to attenuate their capacity to induce demyelination

**Figure 3:**
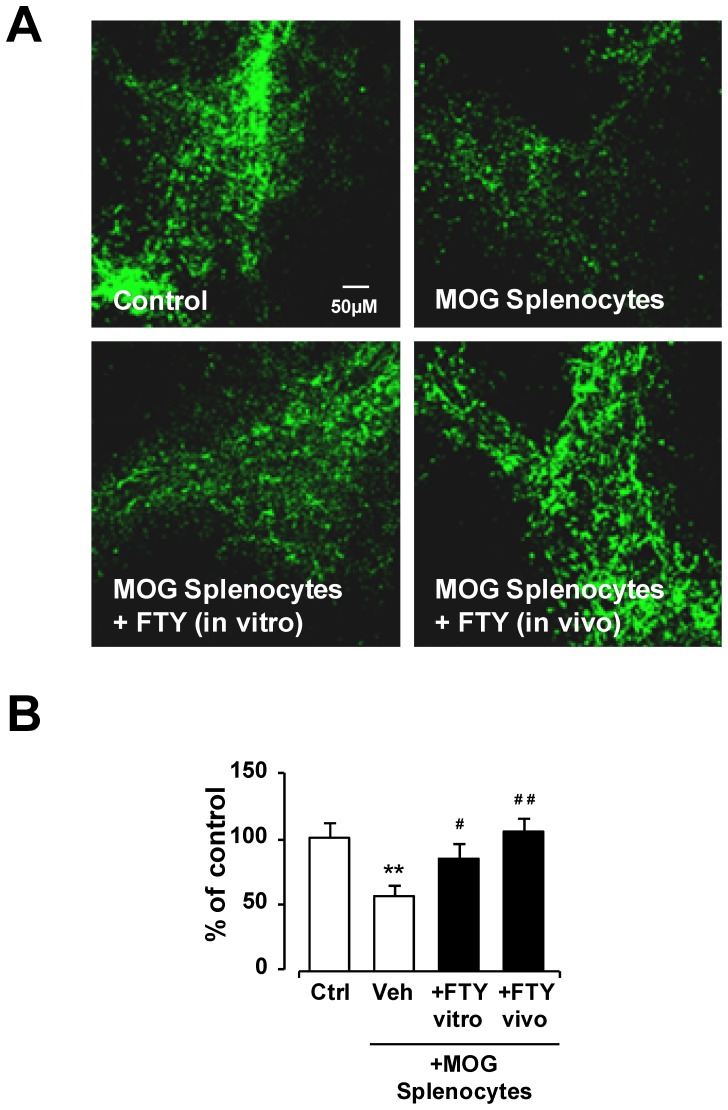
Treatment of pFTY720 in vivo or in vitro reduces MOG reactive splenocyte-induced demyelination isolated from MOG-immunised mice. (A) MOG reactive splenocytes isolated from MOG-immunised animals treated with pFTY720 (8 µg/day, for 10 days) *in vivo* or treated with pFTY720 (1 nM) during re-stimulation *in vitro* with 25 µg/ml MOG prevented demyelination caused by these isolated cells. (B) Bar graph illustrates relative MBP immunoreactivity (i.e. co-localization of MBP and neurofilament) in cerebellar slices compared to no treatment (100% as control) expressed as averages +/− SEM (20 slices per treatment group). There was a significant decrease of MBP staining in slices co-cultured with vehicle treated MOG reactive splenocytes compared to control slices (**p<0.01), which was significantly reveresd by *in vitro* treatment of MOG splenocytes with pFTY720 (^#^p<0.05) or *in vivo* treatment of MOG-immunised mice with pFTY720 (^##^p<0.01). There was no significant difference in MBP staining between these two pFTY720 treatment groups and control slices. One-way ANOVA and Newman-Keuls post-hoc test.

The experiment detailed above shows that cells isolated from MOG-immunised mice treated with FTY720 *in vivo* and then pFTY720 *in vitro* no longer induce demyelination. To elucidate whether pFTY720 had direct effects on the ability of these MOG-reactive cells to demyelinate *in vitro,* cells were isolated from MOG-immunised mouse spleens and activated with MOG_35-55_ peptide (25 µg/ml for 48 h) in the presence or absence of pFTY720 (1 nM) before co-culture with organotypic cerebellar slices. Confocal imaging showed that the addition of vehicle-treated MOG-reactive cells to cerebellar slices induced a significant level of demyelination compared to untreated slice control (55.85%+/−2.4%) (p<0.01, one-way ANOVA and Newmann-Keuls post-hoc test) (**Figure 3**). However, MOG-splenocytes activated in the presence of pFTY720 did not show a significant reduction in myelination (84.7%+/−3.25%) based on MBP expression (**Figure 3**). Therefore, this experiment shows that *in vitro* treatment of MOG-reactive cells with pFTY720, can reduce their ability to induce demyelination.

### In vitro treatment of splenocytes isolated from 2D2 transgenic mice with pFTY720 attenuates demyelination

**Figure 4:**
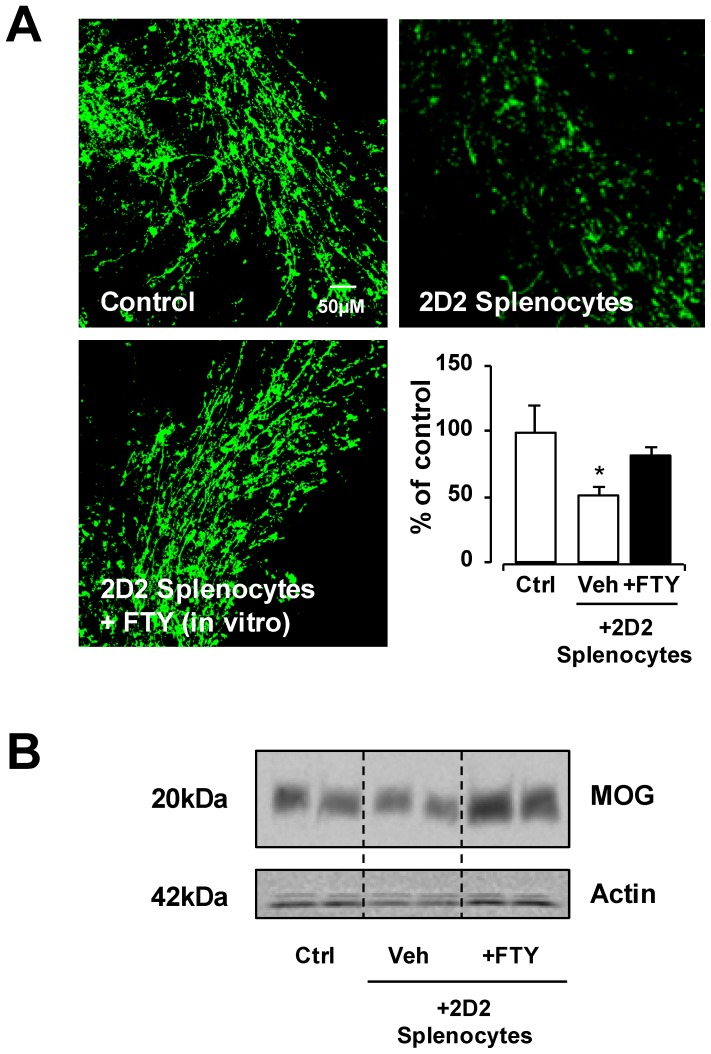
Treatment of pFTY720 in vitro reduces demyelination induced by 2D2-splenocytes. (**A**) 2D2-splenocytes (i.e. splenocytes isolated from 2D2 transgenic mice and stimulated *in vitro* with 25 µg/ml MOG) reduce MBP staining in cerebellar slices. 2D2-splenocytes isolated from 2D2 transgenic mice treated with pFTY720 (1 µM, 48 hrs) during stimulation *in vitro* with 25 µg/ml MOG prevented demyelination caused by these isolated cells. Bar graph illustrates relative MBP immunoreactivity (i.e. co-localization of MBP and neurofilament) in cerebellar slices compared to no treatment (100% as control) expressed as averages +/− SEM (18–24 slices per treatment group). There was a significant decrease of MBP staining in slices co-cultured with vehicle treated 2D2-splenocytes compared to control slices (*p<0.05), which was reduced by pFTY720 treatment; One-way ANOVA and Newman-Keuls post-hoc test. (**B**) Western blot shows decreased MOG protein in 2D2-splenocyte treated slices, with *in vitro* pFTY720 treatment reducing demyelination.

To further confirm that MOG-reactive cells induce demyelination and pFTY720 attenuates this effect, we isolated cells from the spleens of 2D2 transgenic mice (2D2-splenocytes), which transgenically express a T cell receptor for MOG (TCR-MOG) and are thus engineered to be stimulated by MOG. We then examined the effects of pFTY720 on demyelination induced by these MOG-reactive cells in organotypic cerebellar slices. The co-culture of organotypic cerebellar slices with vehicle-treated 2D2-splenocytes induced demyelination, similar to MOG-splenocytes and LPC treatments (**Figure 4A**). MBP staining showed a significant reduction in slices co-cultured with vehicle-treated 2D2-splenocytes (51.6%+/− 6.3%) compared to untreated slice control (p<0.05, one way ANOVA and Newmann-Keuls post-hoc test). Importantly, the *in vitro* treatment of 2D2-splenocytes with pFTY720 (1 µM for 48 h) during re-stimulation with MOG_35-55_ (25 µg/ml for 48 h) before co-culture with organotypic cerebellar slices, attenuated the ability of these cells to induce demyelination (82.5%+/−5.2%) (**Figure 4A**). Western blotting for MOG protein confirmed these findings, and demonstrated a decrease in MOG expression in cerebellar slices co-cultured with vehicle-treated 2D2-splenocytes compared to untreated slice control, whereas pFTY720 attenuated the effects of 2D2-splenocytes to induce demyelination (**Figure 4B**). These data suggest that pFTY720 treatment *in vitro* diminishes the ability of MOG-reactive cells to induce demyelination.

### pFTY720 treatment of 2D2-splenocytes does not alter the T cell phenotype

**Figure 5:**
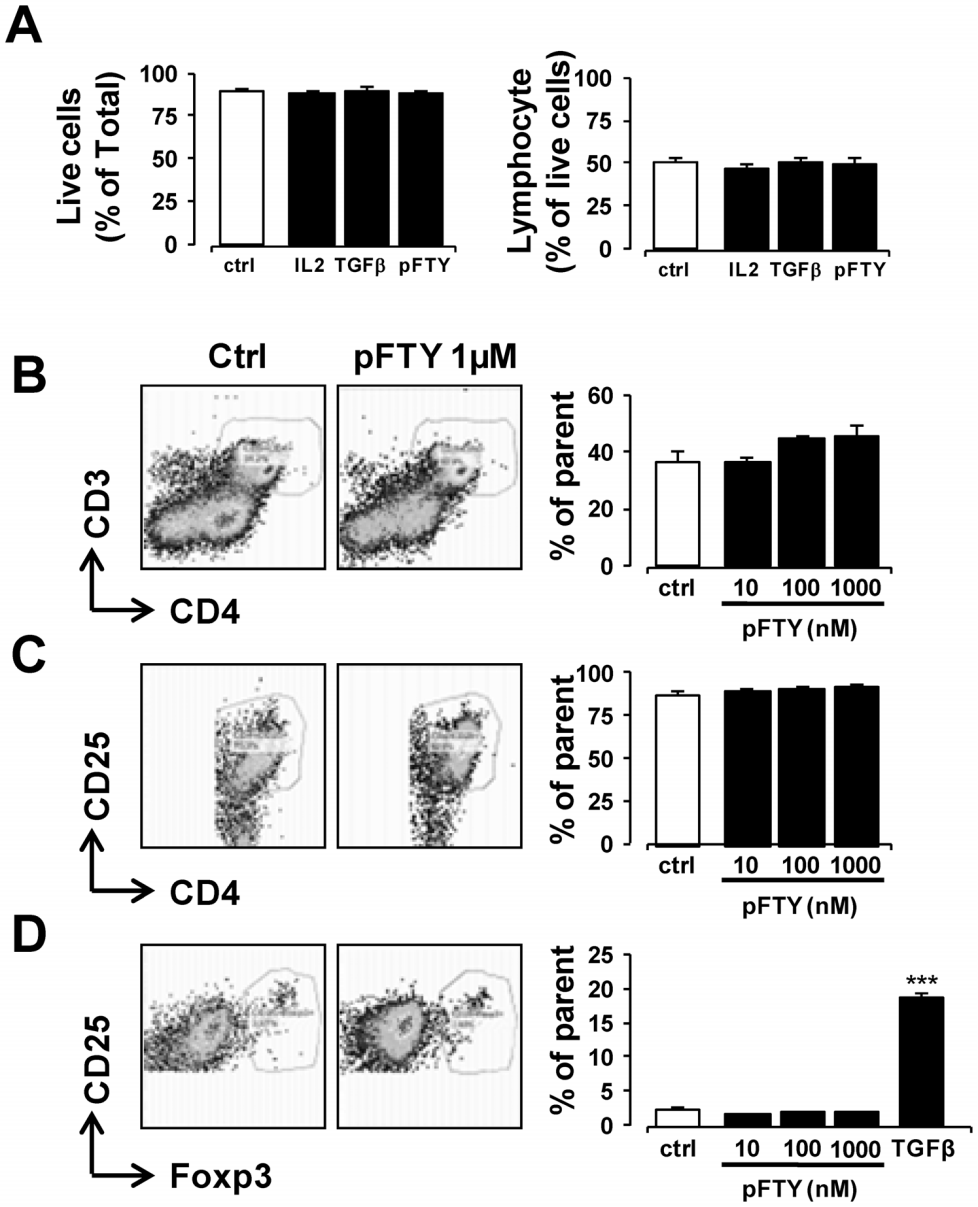
Treatment of pFTY720 in vitro does not alter phenotype of 2D2-splenocytes. Treatment of 2D2-splenocytes with IL2 (20 ng/ml), TGFβ (5 ng/ml) or pFTY720 (10, 100, 1000 nM for 96 hrs, during stimulation with 25 µg/ml MOG_35-55_ peptide did not alter the proportion of (**A**) total live cells as determined by graphing forward scatter height by forward scatter area. In addition the proportion of lymphocytes (lymphocyte gate defined by cell size when forward scatter graphed against side scatter) was not altered by these treatments. (**B**) CD4+ cell (**C**) CD4+/CD25+ cell, or (**D**) CD4+CD25+Foxp3+ Treg cell proportion was not altered by pFTY720 treatment. As a positive control, TGFβ induced a potent and significant increase in CD4+CD25+Foxp3+ expressing Treg cells. The addition of IL2 (20 ng/ml) in conjunction with pFTY720 (10, 100, 1000 nM) did not alter Treg cell frequency (*data not shown*). Bar graphs illustrate values expressed as averages +/− SEM of four independent experiments. Each experiment used splenocytes obtained from four animals. Cell analysis was performed by FACs. Significant difference ***p<0.001 from control; One-way ANOVA and Newman-Keuls post-hoc test.

Studies have proposed that pFTY720 exerts anti-inflammatory effects on T cells, possibly by promoting Treg response, while reducing Th17 cell-mediated inflammation. For example, pFTY720 increases in the proportion of Tregs *in vivo* and *in vitro* [15], [25], [34], while attenuating S1P-induced expansion of Th17 cells and consequent release of IL-17 [35]. Some of these studies have however suggested that pFTY720 reduces numbers of Tregs [18]. In order to ascertain whether *in vitro* treatment of 2D2-splenocytes with pFTY720 altered their phenotype, cells were stimulated with MOG_35-55_ peptide in the presence or absence of this drug. We first investigated the effect of pFTY720 on total cell counts and T cell counts and found that pFTY720 did not alter either of these cell counts (**Figure 5A**). In addition, no significant change in CD4^+^ cell population was observed with pFTY720 treatment (**Figure 5B**). The addition of IL-2, in conjunction with pFTY720, also had no effect on the CD4^+^ percentage (*data not shown*). The effects pFTY720 on specific T cell populations were also examined. No significant change was observed in the proportion of CD4^+^CD25^+^ cells in response to pFTY720 treatment in MOG_35-55_ peptide stimulated 2D2 cells either without (**Figure 5C**) or with IL-2 (*data not shown*). To further examine the effect of pFTY720 on Tregs, cells positive for CD25+Foxp3+ were examined. Treatment of 2D2-splenocytes stimulated with MOG_35-55_ peptide with or without pFTY720 did not alter Treg proportion either in the absence (**Figure 5D**) or presence of IL2 (*data not shown*). In contrast, treatment of 2D2-splenocytes with TGF-β increased the percentage of Tregs by up to 20 fold, as expected (**Figure 5D**). Based on these *in vitro* results, it appears that pFTY720 does not alter Treg populations and is unlikely to explain the effects of this drug in limiting demyelination caused by 2D2-splenocytes in organotypic cerebellar slices.

### pFTY720 significantly reduces pro-inflammatory cytokine release by 2D2-splenocytes in vitro

**Figure 6:**
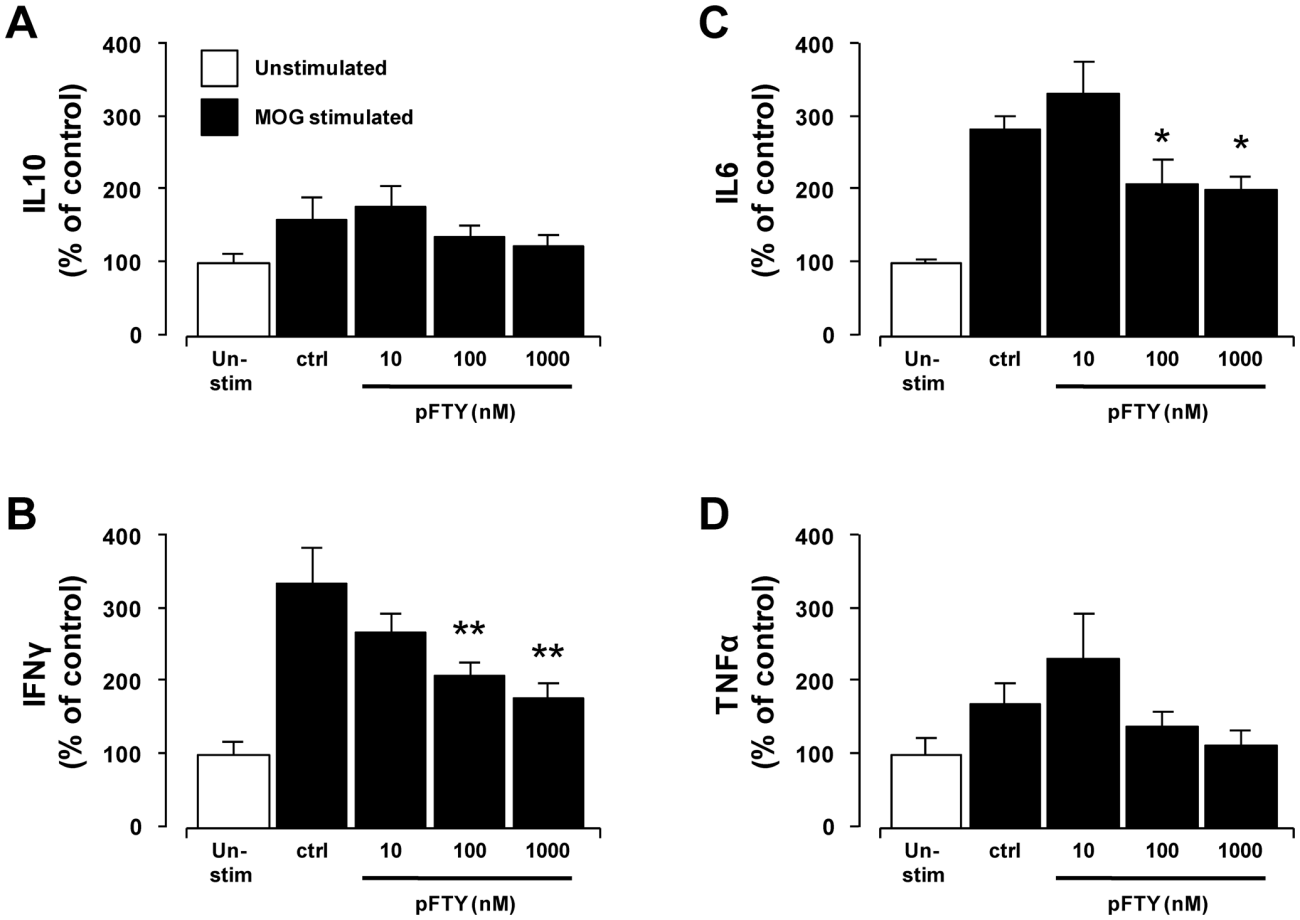
pFTY720 treatment in vitro reduces the release of pro-inflammatory cytokines from 2D2-splenocytes. Treatment with pFTY720 (10, 100, 1000 nM for 96hrs) of 2D2-splenocytes during stimulation with 25 µg/ml MOG_35-55_ peptide did not alter levels of (**A**) IL10 or, (**D**) TNFα but attenuated levels of (**B**) IFNγ, (**C**) IL6. Bar graphs illustrate values expressed as averages +/−SEM of four independent experiments. Each experiment used splenocytes obtained from four animals. Cytokine analysis was performed by ELISA in triplicate. Significant difference *p<0.05, **p<0.01 from control; One-way ANOVA and Newman-Keuls post-hoc test.

IFNγ, TNFα and IL6 are reported as pro-inflammatory cytokines that play a variety of roles in EAE and MS disease state [36]–[38]. In addition, levels of IL10 secreting mononuclear cells have been shown to be reduced in MS sufferers [39], likely indicating impaired anti-inflammatory function. An attenuation of the levels of these pro-inflammatory cytokines, without an impairment of anti-inflammatory cytokine release, would likely have numerous beneficial effects in myelination state. Previous studies have shown that S1P attenuates the production of IFNγ by splenic CD4^+^ T cells, and this effect is likely mediated via S1PR1, although the direct effects of pFTY720 were not tested at that time [35], [40]. Moreover S1PRs also regulate the release of TNFα and IL6 from cells types such as dendritic cells, macrophages and microglia [41]–[43]. Here we found pFTY720 did not significantly alter IL10 levels in 2D2-cells stimulated with MOG_35-55_ peptide (**Figure 6A**). In contrast, pFTY720 reduced the levels of IFNγ in a concentration dependent manner, where 2D2-cells stimulated with MOG_35-55_ peptide in the presence of pFTY720 (1 µM) displayed a significant reduction in levels of IFNγ (122.2% +/−20%) compared to MOG-stimulated control (304% +/- 50.8%, p<0.01, one-way ANOVA and Newmann-Keuls post-hoc test) (**Figure 6B**). pFTY720 (1 µM) also significantly reduced the levels of IL6 (198%+/−18.8%), compared to MOG-stimulated control (280.9% +/−18.5%, p<0.05, one way ANOVA and Newmann-Keuls post-hoc test) (**Figure 6C**). The effect of pFTY720 on levels of TNFα in MOG_35-55_ peptide activated 2D2-cells was moderate, with a trend reduction in levels of TNFα induced by pFTY720, which was not significant (**Figure 6D**). While we have not specifically investigated immune cell subtypes, these data suggest that FTY720 does not cause overt cellular apoptosis when splenocytes are treated *in vitro* with this compound (**Figure 5A**) that would account for the decreased cytokine production. Taken together, these data suggest that pFTY720 may attenuate demyelination induced by MOG-reactive cells (isolated from the spleens of 2D2-transgenic animals) by limiting levels of pro-inflammatory molecules.

## Discussion

We and others have previously reported that pFTY720 promotes remyelination and attenuates demyelination in organotypic slice cultures, which contain immune cells [21]–[24]. In these studies LPC was used to induce demyelination, however, a concern using this agent is that the mechanism by which LPC induces demyelination, *in vitro* or *in vivo*, is not fully clear. Therefore, in the current study we aimed to further these previous findings using a splenocyte cell culture-mediated demyelination model. In particular, we determined if pFTY720 could limit demyelination in organotypic slice cultures by directly acting on exogenously applied immune cells. Here, we found treatment of MOG-reactive splenocytes with pFTY720 was sufficient to attenuate demyelination induced by these cells. We also found that pFTY720 decreased the levels of pro-inflammatory cytokines, such as IFNγ and IL6 (and likely others) in immune cells that may potentially play a role by which this compound limits demyelination and/or promotes remyelination.

In addition to these findings, there were some important technical features of this study which are noteworthy. Firstly, we considered the possibility that pFTY720 added to the splenocyte cultures may be taken up by these cells and even after washing, this compound may persist and be carried over to the cerebellar culture, where we have shown it to have a direct effect on myelination [22]. This possibility, however, appears unlikely given previous studies showing that the phosphorylated from pFTY720 does not permeate lymphocytes, must be de-phosphorylated in order to enter the intra-cellular space, and then requires phosphorylation back to pFTY720 before leaving the cell and acting on receptors [44]. Moreover when 5 ×10^6^ cells are pre-treated with 1 µM FTY720 for 1 hr, these were shown to contain 0.1–0.2 nmol pFTY720 [44]. In our *in vitro* studies we used pFTY720, which in this from does not permeate lymphocytes and we also used 100 times less the number of cells (1×10^4^), which accordingly would contain <1–2 pmol of pFTY720 and release even less.

Secondly, while it is well accepted that pFTY720 causes receptor internalisation, with little effect on S1PR1 expression at the mRNA level, the fate of the internalised S1PR1 function remains unclear, namely, continued signalling [45] and/or receptor downregulation [46]–[47]. We and others have shown S1PR1 receptor internalisation occurs in astrocytes [30], [48] and also in HUVEC cells [45], as well as in transfected murine B-cell line (WEHI231) [6], and a stable HEK293 cells expressing epotope tagged-S1PR1 [46]. Furthermore, the internalisation of S1PRs in T cells, by pFTY720, is suggested to make them insensitive to the gradients of S1P that exist between the lymphoid organs and the peripheral circulation, which inhibits the mobility of activated T cell and prevents them from exiting lymph nodes [6], [13]. In knockout and cell transfer approaches, this activity has been well characterised *in vivo* and explains much of the beneficial effect of this drug in MS. To date, however, the lack of good S1PR1 antibodies has limited directly investigating pFTY720-mediated S1PR1 internalisation in either mouse or human T cells. Thus, in the current study, we have not investigated S1PR1 internalisation in T cell, although we used similar concentrations of pFTY720 to treat splenocyte cultures as previously reported to cause S1PR1 receptor internalisation [6], [45]–[48]. We propose, therefore, that pFTY720 is inducing S1PR1 receptor internalisation in our current study and in other previous reports, and likely inhibiting immune cell function, which is also supported partially by findings that pFTY720 suppresses S1P-mediated development of Th17 cells and levels of IL17 [35].

Thirdly, we used splenocyte cultures that, in addition to T cells, likely include cells such as macrophages, B cells, natural killer cells and dendritic cells. Our analysis of these splenocyte cultures showed an approximate 60% lymphocyte population, of which more than 40% were CD4+ T cells. We note the cytokines analysed in our study, IL10, IL6, IFNγ and TNFα can be produce by cells other than CD4+ T cells, where S1PR activation has been shown to alter cytokine levels in, for example, macrophages (attenuating levels of TNFα, MCP1 and IL6) [42] and dendritic cells (reducing levels of IL12 and increasing IL10) [49]. Furthermore, pFTY720 treated dendritic cells show reduced ability to activate T cells, which in turn produce less IFNγ and more IL4 compared to when activated with untreated dendritic cells, indicating a shift from a pro-inflammatory Th1 to an anti-inflammatory Th2 [41], [49]–[50]. While these reports suggest that studies investigating whether individual immune cell subtypes can induce demyelination and if pFTY720 can reverse these effects are worthy, such isolated cellular effects are unlikely germane in an *in vivo* milieu. Our data showing that pFTY720 can protect against demyelination induced by a mixture of immune cells is thus, we consider, relevant moreso to an *in vivo* setting.

Fourthly, in this current study, we used splenocyte cultures from 2D2 trangenic animals to induce demyelination. The cells isolated from these 2D2 mice contain T cells engineered to express a T cell receptor activated by MOG (TCR-MOG) and can thus be stimulated by MOG to specifically generate MOG-reactive T cells. We again opted for using a splenocyte culture approach assuming it would be important to generate MOG-reactive T cells in a mixture of cells rather than isolating this individual cell subtype and devoiding it from environmental support. Furthermore, while we consider these MOG-reactive transplanted splenocytes to target MOG-expressing cells (i.e. oligodendrocytes), other cells in this slice culture such as astrocyte, microglia and likely a variety of already resident immune cells are also potentially indirectly targeted and play substantial role(s). The selective cellular targeting of these organotypic slice resident cells would be technically challenging, and further compromise experiments aiming to investigate the effect of individual immune cell subtypes on demyelination.

Fithly, pFTY720 has been purported to induce a phenotypic change in some T cells that causes them to take on a Treg function [15], [34], [35]. In our current study we aimed to further examine if pFTY720 altered numbers of Tregs and, for this purpose, we used a mixed population of splenocytes isolated from 2D2 transgenic mice. We found that *in vitro* treatment of these cells with pFTY720 did not alter T cell phenotype; specifically there was no effect on CD4 and Foxp3 expressing Tregs. In contrast, the positive control, TGFβ treatment caused a large increase in this population of cells, as expected. Notably, previous studies have utilised T cell populations enriched for CD4^+^CD25^−^ cells in order to demonstrate an increase in Treg proportion induced by pFTY720 and moreover some studies have suggested that pFTY720 reduces numbers of Tregs [18]. The variations between these and our studies may be potentially explained by the types of cultures used for these experiments, that is, mixed splenocyte or enriched populations.

Lastly, in addition to pFTY720 altering T cell transmigration and possible lymphocyte phenotype (although this requires further corroboration), pFTY720 can alter cytokine release from immune cells [49]–[51]. We choose to examine the effects of pFTY720 on the levels of IFNγ in MOG-reactive cells given that this cytokine regulates Th1 cell differentiation, CD8^+^ cell-induced cell death and microglial recruitment in MS [35], [36], [52]–[55], that S1P attenuates the production of IFNγ by splenic CD4 T cells [40] and that effect of pFTY720 on IFNγ was not examined in these reports [35], [40]. In addition, the levels of IL6 were also tested as this cytokine desentitises effector T cells to regulation by Tregs in MS, as well as being elevated in the serum and CSF of MS patients [37], [56]. We also examined the effects of pFTY720 on the levels of TNFα in MOG-reactive cells as levels of this cytokine are elevated in CSF, serum and post-mortem in the brain lesions of MS patients and may be linked to severity of lesions [57], despite anti-TNFα therapies not being beneficial in MS [58], [59]. We found that *in vitro* treatment with pFTY720 caused a significant concentration-dependent decrease in IFNγ and IL6 in activated 2D2-splenocytes, while having moderate effect on TNFα and no effect on IL10. This appears consistent with the idea that pFTY720 likely causes a broad attenuation in the levels of pro-inflammatory cytokines, in agreement with our previous findings [22].

In conclusion, therefore, this study suggests that pFTY720 treatment of MOG-reactive splenocytes (either from MOG-immunised mice or 2D2-transgenic mice) can reduce the release of pro-inflammatory cytokines and can reduce the ability of these cells to induce demyelination. These results may suggest that, in addition to preventing transmigration of immune cells into the CNS and having direct effects on neuronal and glia cells, pFTY720 may also attenuate pro-inflammatory signals in immune cells that have already passed into the CNS prior to treatment. In closing, these mechanisms, in concert, may thus better explain the therapeutic efficacy of pFTY720 in relapsing-remitting MS.

## References

[1] Lee M, Van Brocklyn JR, Thangada S, Liu CH, Hand AR, et al (1998) Sphingosine-1-Phosphate as a Ligand for the G Protein-Coupled Receptor EDG-1. Science (80-) 279: 1552–1555. 10.1126/science.279.5356.1552 9488656

[2] Yamazaki Y, Kon J, Sato K, Tomura H, Sato M, et al (2000) Edg-6 as a putative sphingosine 1-phosphate receptor coupling to Ca(2+) signaling pathway. Biochem Biophys Res Commun 268: 583–589. 10679247 10.1006/bbrc.2000.2162

[3] Im D-S, Heise CE, Ancellin N, O'Dowd BF, Shei G-J, et al (2000) Characterization of a novel sphingosine 1-phosphate receptor, Edg-8. J Biol Chem 275: 14281–14286. 10799507 10.1074/jbc.275.19.14281

[4] Okamoto H, Takuwa N, Yatomi Y, Gonda K, Shigematsu H, et al (1999) EDG3 is a functional receptor specific for sphingosine 1-phosphate and sphingosylphosphorylcholine with signaling characteristics distinct from EDG1 and AGR16. Biochem Biophys Res Commun 260: 203–208. 10381367 10.1006/bbrc.1999.0886

[5] An S, Bleu T, Huang W, Hallmark OG, Coughlin SR, et al (1997) Identification of cDNAs encoding two G protein-coupled receptors for lysosphingolipids. FEBS Lett 417: 279–282. 9409733 10.1016/s0014-5793(97)01301-x

[6] Matloubian M, Lo CG, Cinamon G, Lesneski MJ, Xu Y, et al (2004) Lymphocyte egress from thymus and peripheral lymphoid organs is dependent on S1P receptor 1. Nature 427 355–60. 14737169 10.1038/nature02284

[7] Kappos L, Radue E-W, O'Connor P, Polman C, Hohlfeld R, et al (2010) A Placebo-Controlled Trial of Oral Fingolimod in Relapsing Multiple Sclerosis. N Engl J Med 362: 387–401. 20089952 10.1056/NEJMoa0909494

[8] Taha T, Hannun Y, Obeid L (2006) Sphingosine kinase: biochemical and cellular regulation and role in disease. J Biochem Mol Biol 39: 113–131. 16584625 10.5483/bmbrep.2006.39.2.113

[9] Ogretmen B, Hannun YA (2004) Biologically active sphingolipids in cancer pathogenesis and treatment. Nat Rev Cancer 4: 604–616. 15286740 10.1038/nrc1411

[10] Zemann B, Kinzel B, Müller M, Reuschel R, Mechtcheriakova D, et al (2006) Sphingosine kinase type 2 is essential for lymphopenia induced by the immunomodulatory drug FTY720. Blood 107: 1454–1458. 16223773 10.1182/blood-2005-07-2628

[11] Kharel Y, Lee S, Snyder AH, Sheasley-O'neill SL, Morris MA, et al (2005) Sphingosine kinase 2 is required for modulation of lymphocyte traffic by FTY720. J Biol Chem 280: 36865–36872. 16093248 10.1074/jbc.M506293200

[12] Brinkmann V, Davis MD, Heise CE, Albert R, Cottens S, et al (2002) The immune modulator FTY720 targets sphingosine 1-phosphate receptors. J Biol Chem 277: 21453–21457. 11967257 10.1074/jbc.C200176200

[13] Mandala S, Hajdu R, Bergstrom J, Quackenbush E, Xie J, et al (2002) Alteration of lymphocyte trafficking by sphingosine-1-phosphate receptor agonists. Science 296: 346–349. 11923495 10.1126/science.1070238

[14] Sun Y, Wang W, Shan B, Di J, Chen L, et al (2011) FTY720-induced conversion of conventional Foxp3- CD4+ T cells to Foxp3+ regulatory T cells in NOD mice. Am J Reprod Immunol 66: 349–362. 21623989 10.1111/j.1600-0897.2011.01010.x

[15] Sehrawat S, Rouse BT (2008) Anti-inflammatory effects of FTY720 against viral-induced immunopathology: role of drug-induced conversion of T cells to become Foxp3+ regulators. J Immunol 180: 7636–7647. 18490766 10.4049/jimmunol.180.11.7636

[16] Fontenot J, Gavin M, Rudensky A (2003) Foxp3 programs the development and function of CD4+ CD25+ regulatory T cells. Nat Immunol 4: 330–336. 12612578 10.1038/ni904

[17] Fantini MC, Dominitzki S, Rizzo A, Neurath MF, Becker C (2007) In vitro generation of CD4+ CD25+ regulatory cells from murine naive T cells. Nat Protoc 2: 1789–1794. 17641646 10.1038/nprot.2007.258

[18] Wolf AM, Eller K, Zeiser R, Gerlach U V, Sixt M, et al (2009) The Sphingosine 1-phosphate Receptor Agonist FTY720 Potently Inhibits Regulatory T Cell Proliferation In Vitro and In Vivo. J Immunol 183: 3751–3760. 19692647 10.4049/jimmunol.0901011

[19] Dev KK, Mullershausen F, Mattes H, Kuhn RR, Bilbe G, et al (2008) Brain sphingosine-1-phosphate receptors: implication for FTY720 in the treatment of multiple sclerosis. Pharmacol Ther 117: 77–93. 17961662 10.1016/j.pharmthera.2007.08.005

[20] Pritchard AJ, Dev KK (2013) The role of S1P receptors in the treatment of demyelinating diseases. Future Neurology 8: 487–489.

[21] Miron VE, Ludwin SK, Darlington PJ, Jarjour AA, Soliven B, et al (2010) Fingolimod (FTY720) enhances remyelination following demyelination of organotypic cerebellar slices. Am J Pathol 176: 2682–2694. 20413685 10.2353/ajpath.2010.091234 PMC2877831

[22] Sheridan GK, Dev KK (2012) S1P1 receptor subtype inhibits demyelination and regulates chemokine release in cerebellar slice cultures. Glia 60: 382–392. 22108845 10.1002/glia.22272

[23] Ling C, Verbny Y, Banks M (2008) In situ activation of antigen-specific CD8+ T cells in the presence of antigen in organotypic brain slices. J Immunol 180: 8393–8399. 18523307 10.4049/jimmunol.180.12.8393 PMC3338102

[24] Prodinger C, Bunse J, Krüger M (2011) Schiefenhövel F, Brandt C, et al (2011) CD11c-expressing cells reside in the juxtavascular parenchyma and extend processes into the glia limitans of the mouse nervous system. Acta Neuropathol 121: 445–458. 21076838 10.1007/s00401-010-0774-y

[25] Kim HJ, Miron VE, Dukala D, Proia RL, Ludwin SK, et al (2011) Neurobiological effects of sphingosine 1-phosphate receptor modulation in the cuprizone model. FASEB J 25: 1509–1518. 21248243 10.1096/fj.10-173203 PMC3079302

[26] Jaillard C, Harrison S, Stankoff B, Aigrot MS, Calver AR, et al (2005) Edg8/S1P5: an oligodendroglial receptor with dual function on process retraction and cell survival. J Neurosci 25: 1459–1469. 15703400 10.1523/JNEUROSCI.4645-04.2005 PMC6726002

[27] Hu Y, Lee X, Ji B, Guckian K, Apicco D, et al (2011) Sphingosine 1-phosphate receptor modulator fingolimod (FTY720) does not promote remyelination in vivo. Mol Cell Neurosci 48: 72–81. 21740973 10.1016/j.mcn.2011.06.007

[28] Jackson SJ, Giovannoni G, Baker D (2011) Fingolimod modulates microglial activation to augment markers of remyelination. J Neuroinflammation 8: 76. 21729281 10.1186/1742-2094-8-76 PMC3152910

[29] Bettelli E, Pagany M, Weiner HL, Linington C, Sobel RA, et al (2003) Myelin oligodendrocyte glycoprotein-specific T cell receptor transgenic mice develop spontaneous autoimmune optic neuritis. J Exp Med 197: 1073–1081. 12732654 10.1084/jem.20021603 PMC2193967

[30] Healy LM, Sheridan GK, Pritchard AJ, Rutkowska A, Mullershausen F, et al (2013) Pathway specific modulation of S1P1 receptor signalling in rat and human astrocytes. Br J Pharmacol 169: 1114–1129. 23587004 10.1111/bph.12207 PMC3696333

[31] Yang Y, Lewis R, Miller RH (2011) Interactions between oligodendrocyte precursors control the onset of CNS myelination. Dev Biol. 350: 127–38. 21144846 10.1016/j.ydbio.2010.11.028 PMC3032606

[32] Bin JM, Leong SY, Bull SJ, Antel JP, Kennedy TE (2012) Oligodendrocyte precursor cell transplantation into organotypic cerebellar shiverer slices: a model to study myelination and myelin maintenance. PLoS One. 7: e41237. 22911763 10.1371/journal.pone.0041237 PMC3401123

[33] Shrestha R, Millington O, Brewer J, Dev KK, Bushell TJ (2014) Lymphocyte-mediated neuroprotection in in vitro models of excitotoxicity involves astrocytic activation and the inhibition of MAP kinase signalling pathways. Neuropharmacology 76: 184–93. 23831681 10.1016/j.neuropharm.2013.06.025

[34] Daniel C, Sartory N, Zahn N, Geisslinger G, Radeke HH, et al (2007) FTY720 ameliorates Th1-mediated colitis in mice by directly affecting the functional activity of CD4+CD25+ regulatory T cells. J Immunol 178: 2458–2468. 17277153 10.4049/jimmunol.178.4.2458

[35] Liao J-J, Huang M-C, Goetzl EJ (2007) Cutting edge: Alternative signaling of Th17 cell development by sphingosine 1-phosphate. J Immunol 178: 5425–5428. 17442922 10.4049/jimmunol.178.9.5425

[36] Murphy AC, Lalor SJ, Lynch MA, Mills KHG (2010) Infiltration of Th1 and Th17 cells and activation of microglia in the CNS during the course of experimental autoimmune encephalomyelitis. Brain Behav Immun 24: 641–651. 20138983 10.1016/j.bbi.2010.01.014

[37] Schneider A, Long SA, Cerosaletti K, Ni CT, Samuels P, et al (2013) In active relapsing-remitting multiple sclerosis, effector T cell resistance to adaptive T(regs) involves IL-6-mediated signaling. Sci Transl Med 5: 170ra15. 10.1126/scitranslmed.3004970 23363979

[38] Akassoglou K, Bauer J, Kassiotis G, Pasparakis M, Lassmann H, et al (1998) Oligodendrocyte apoptosis and primary demyelination induced by local TNF/p55TNF receptor signaling in the central nervous system of transgenic mice: models for multiple sclerosis with primary oligodendrogliopathy. Am J Pathol 153: 801–813. 9736029 10.1016/S0002-9440(10)65622-2 PMC1853008

[39] Ozenci V, Kouwenhoven M, Huang YM, Xiao B, Kivisäkk P, et al (1999) Multiple sclerosis: levels of interleukin-10-secreting blood mononuclear cells are low in untreated patients but augmented during interferon-beta-1b treatment. Scand J Immunol 49: 554–561. 10320650 10.1046/j.1365-3083.1999.00546.x

[40] Dorsam G, Graeler MH, Seroogy C, Kong Y, Voice JK, et al (2003) Transduction of multiple effects of sphingosine 1-phosphate (S1P) on T cell functions by the S1P1 G protein-coupled receptor. J Immunol 171: 3500–3507. 14500646 10.4049/jimmunol.171.7.3500

[41] Oz-Arslan D, Ruscher W, Myrtek D, Ziemer M, Jin Y, et al (2006) IL-6 and IL-8 release is mediated via multiple signaling pathways after stimulating dendritic cells with lysophospholipids and immunoblot analyses indicate that immature as. J Leukoc Biol 80: 287–297. 16769764 10.1189/jlb.1205751

[42] Potì F, Gualtieri F, Sacchi S, Weiβen-Plenz G, Varga G, et al (2013) KRP-203, sphingosine 1-phosphate receptor type 1 agonist, ameliorates atherosclerosis in LDL-R-/- mice. Arterioscler Thromb Vasc Biol 33: 1505–1512. 23640484 10.1161/ATVBAHA.113.301347

[43] Noda H, Takeuchi H, Mizuno T, Suzumura A (2013) Fingolimod phosphate promotes the neuroprotective effects of microglia. J Neuroimmunol 256: 13–18. 23290828 10.1016/j.jneuroim.2012.12.005

[44] Sensken S, Bode C, Gräler M (2009) Accumulation of fingolimod (FTY720) in lymphoid tissues contributes to prolonged efficacy. . J. Pharmacol. Exp. Ther 328: 963–969. 19074680 10.1124/jpet.108.148163

[45] Mullershausen F, Zecri F, Cetin C, Billich A, Guerini D, et al (2009) Persistent signaling induced by FTY720-phosphate is mediated by internalized S1P1 receptors. Nat Chem Biol 5: 428–435. 19430484 10.1038/nchembio.173

[46] Oo ML, Chang SH, Thangada S, Wu MT, Rezaul K, et al (2011) Engagement of S1P1-degradative mechanisms leads to vascular leak in mice. J Clin Invest 121: 2290–2300. 21555855 10.1172/JCI45403 PMC3104755

[47] Oo ML, Thangada S, Wu MT, Liu CH, Macdonald TL, et al (2007) Immunosuppressive and anti-angiogenic sphingosine 1-phosphate receptor-1 agonists induce ubiquitinylation and proteasomal degradation of the receptor. J Biol Chem 282: 9082–9089. 17237497 10.1074/jbc.M610318200

[48] Choi JW, Gardell SE, Herr DR, Rivera R, Lee CW, et al (2011) FTY720 (fingolimod) efficacy in an animal model of multiple sclerosis requires astrocyte sphingosine 1-phosphate receptor 1 (S1P1) modulation. Proc Natl Acad Sci U S A. 108: 751–756. 21177428 10.1073/pnas.1014154108 PMC3021041

[49] Müller H, Hofer S, Kaneider N, Neuwirt H, Mosheimer B, et al (2005) The immunomodulator FTY720 interferes with effector functions of human monocyte-derived dendritic cells. Eur J Immunol 35: 533–545. 15657952 10.1002/eji.200425556

[50] Idzko M, Panther E, Corinti S, Morelli A, Ferrari D, et al (2002) Sphingosine 1-phosphate induces chemotaxis of immature and modulates cytokine-release in mature human dendritic cells for emergence of Th2 immune responses. FASEB J 16: 625–627. 11919175 10.1096/fj.01-0625fje

[51] Hwang SJ, Kim JH, Kim HY, Kim S, Chung DH (2010) FTY720, a sphingosine 1-phosphate receptor modulator, inhibits CD1d-restricted NKT cells by suppressing cytokine production but not migration. Lab Investig 90: 9–19. 19823172 10.1038/labinvest.2009.109

[52] Sepulcre J, Sanchez-Ibarrola A, Moreno C, de Castro P (2005) Association between peripheral IFN-gamma producing CD8+ T-cells and disability score in relapsing-remitting multiple sclerosis. Cytokine 32: 111–116. 16246570 10.1016/j.cyto.2005.08.005

[53] Buntinx M, Ameloot M, Steels P, Janssen P, Medaer R, et al (2002) Interferon-gamma-induced calcium influx in T lymphocytes of multiple sclerosis and rheumatoid arthritis patients: a complementary mechanism for T cell activation? J Neuroimmunol 124: 70–82. 11958824 10.1016/s0165-5728(01)00495-7

[54] Panitch H, Haley A, Hirsch R, Johnson K (1987) Exacerbations of multiple sclerosis in patients treated with gamma interferon. Lancet 329: 893–895. 10.1016/s0140-6736(87)92863-7 2882294

[55] Aloisi F, Ria F, Adorini L (2000) Regulation of T-cell responses by CNS antigen-presenting cells: different roles for microglia and astrocytes. Immunol Today 21: 141–147. 10689302 10.1016/s0167-5699(99)01512-1

[56] Stelmasiak Z, Koziol-Montewka M, Dobosz B, Rejdak K, Bartosik-Psujek H, et al (2000) Interleukin-6 concentration in serum and cerebrospinal fluid in multiple sclerosis patients. Med Sci Monit 6: 1108–1108. 11208463

[57] McCoy MK, Tansey MG (2008) TNF signaling inhibition in the CNS: implications for normal brain function and neurodegenerative disease. J Neuroinflammation 5: 45. 18925972 10.1186/1742-2094-5-45 PMC2577641

[58] Van Oosten BW, Barkhof F, Truyen L, Boringa JB, Bertelsmann FW, et al (1996) Increased MRI activity and immune activation in two multiple sclerosis patients treated with the monoclonal anti-tumor necrosis factor antibody cA2. Neurol 47 1531–1534. 10.1212/wnl.47.6.1531 8960740

[59] Sicotte NL, Voskuhl RR (2001) Onset of multiple sclerosis associated with anti-TNF therapy. Neurology 57: 1885–1888. 11723281 10.1212/wnl.57.10.1885

